# Development of Near-Isogenic Lines in a Parthenogenetically Reproduced Thrips Species, *Frankliniella occidentalis*

**DOI:** 10.3389/fphys.2017.00130

**Published:** 2017-03-13

**Authors:** Guangdi Yuan, Yanran Wan, Xiaoyu Li, Bingqing He, Youjun Zhang, Baoyun Xu, Shaoli Wang, Wen Xie, Xuguo Zhou, Qingjun Wu

**Affiliations:** ^1^Institute of Vegetables and Flowers, Chinese Academy of Agricultural SciencesBeijing, China; ^2^Department of Entomology, University of KentuckyLexington, KY, USA

**Keywords:** *Frankliniella occidentalis*, near-isogenic lines, insecticide resistance, spinosad, ISSR

## Abstract

Although near-isogenic lines (NILs) can standardize genetic backgrounds among individuals, it has never been applied in parthenogenetically reproduced animals. Here, through multiple rounds of backcrossing and spinosad screening, we generated spinosad resistant NILs in the western flower thrips, *Frankliniella occidentalis* (Pergande) (Thysanoptera: Thripidae), with a haplo-diploid reproduction system. The resultant *F. occidentalis* NIL-R strain maintained a resistance ratio over 30,000-fold, which was comparable to its parental resistant strain, Spin-R. More importantly, *F. occidentalis* NIL-R shared 98.90% genetic similarity with its susceptible parental strain Ivf03. By developing this toolset, we are able to segregate individual resistance and facilitate the mechanistic study of insecticide resistances in phloem-feeding arthropods, a group of devastating pest species reproducing sexually as well as asexually.

## Introduction

Near-isogenic lines (NILs) are strains which genetic makeups are identical except for few specific locations or genetic loci (Muehlbauer et al., 1988; Young et al., 1988). The use of NILs minimizes the effects caused by different genetic backgrounds to avoid epistatic gene interactions that may occur in outbred strains (Zhu et al., 2015). NILs have been extensively used in gene mapping, gene function analysis, and the development of new cultivars in plants (Brouwer and St Clair, 2004; von-Korff et al., 2004; Edwards et al., 2005; Loudet et al., 2005; Blanco et al., 2006). In insects, NILs are used predominantly in the context of insecticide or stress resistance, including the inheritance of resistance (Roush and Wolfenbarger, 1985; White and Bell, 1988), relative fitness between resistant and susceptible populations (Jiang et al., 2007), discovery of molecular markers (Mu et al., 2005a), and identification of resistance genes (Hardstone et al., 2010). Classic NILs construction methods, such as backcross inbred lines (BILs) and recombinant inbred lines (RILs), are applicable exclusively to amphigenetic insects, including fruit fly, beet armyworm, diamondback moth, and silkworm (Pierce and Lucchesi, 1981; Mu et al., 2004; Yuan et al., 2010; Zhu et al., 2015). Social animals, such as honeybees, ants, and termites (Tucker, 1958; Bell, 1982; Matsuura, 2010), and phloem-sucking arthropods, including aphids, thrips, whiteflies, and spider mites (Helle and Bolland, 1967; Dixon, 1987; Bink-Moenen and Mound, 1990; Murai, 1990), reproduce both sexually and asexually. Therefore, a novel construction method is needed to establish NILs in these animals.

The western flower thrips, *Frankliniella occidentalis* (Pergande) (Thysanoptera: Thripidae), has a haplo-diploid reproduction system, in which females and males are developed from fertilized and unfertilized eggs, respectively. Their unmated females produce only male offspring, whereas mated females produce both males and females (Bryan and Smith, 1956). As a polyphagous insect, *F. occidentalis* causes extensive damages to a wide range of crops by feeding, egg laying, and vectoring of plant tospoviruses (Sakimura, 1962). Its small body size, high fecundity, and short life cycle facilitate *F. occidentalis* to develop resistance to wide range of pesticides, including organochlorines, organophosphates, carbamates, pyrethroids, and spinosad (Immaraju et al., 1992; Brødsgaard, 1994; Martin et al., 1994; Robb et al., 1995; Zhao et al., 1995; Bielza et al., 2007b). Although spinosad has been the most effective synthetic pesticide against *F. occidentalis* (Thompson et al., 2000; Williams et al., 2003), cases of spinosad resistance and management failure have been reported globally (Loughner et al., 2005; Bielza et al., 2007b; Herron and James, 2007). In China, *F. occidentalis* has developed over 100-fold resistance to spinosad in the field, and caused damages to regional agriculture (Wan et al., 2016).

A genetic linkage experiment has demonstrated that spinosad resistance in *F. occidentalis* was controlled by a single autosomal recessive gene and was not influenced by maternal effects (Bielza et al., 2007a). To study this recessive trait, the establishment of a spinosad resistant NIL sharing the same genetic background with susceptible *F. occidentalis* is critical for the mechanistic and genetic understanding of spinosad resistance. During the development of NILs, a series of molecular markers were used in marker-assisted selection (MAS) or examining the genetic background of backcross derivatives, like restriction fragment length polymorphism (RFLP) (Shen et al., 2001), Simple Sequence Repeat (SSR) (Wang et al., 2009) and inter-simple sequence repeat (ISSR) (Zhu et al., 2015). Because of a higher frequency of polymorphism, a greater repeatability and a lower cost, ISSR was chosen for this study (Godwin et al., 1997). Amplification of ISSR-PCR, however, is affected by the concentration of each PCR ingredient, and different combinations. Therefore, an orthogonal experiment was designed to optimize the ISSR-PCR reaction system in *F. occidentalis* to obtain as many polymorphic bands as possible to achieve an accurate measurement of near-isogenicity.

## Materials and methods

### Frankliniella occidentalis strains

One spinosad-susceptible (Ivf03) and one spinosad-resistant strain (Spin-R) of *F. occidentalis* were used. The susceptible Ivf03 strain was collected in a greenhouse at the Institute of Vegetables and Flowers, Chinese Academy of Agricultural Sciences (CAAS) in Beijing, China in 2003, and was subsequently maintained on bean pods without exposure to any insecticides (Zhang et al., 2007). The resistant Spin-R strain was derived from Ivf03 by exposing thrips to bean pods treated with formulated spinosad (Spinoace, Dow AgroSciences China Co., Ltd., China), and the resistance ratio of Spin-R was over >10^4^-fold (Hou et al., 2013). Both strains were kept separately but under the same conditions, i.e., 27 ± 1°C and 50% relative humidity.

### Toxicity bioassay

A leaf-tube residual film method (Hou et al., 2014) was adopted to determine the diagnostic concentration between Ivf03 and Spin-R strains, and to calculate the resistance ratios of the offspring. At least five concentrations were used in each bioassay with four replicates for each concentration and 15 adult females for each replication. The treated thrips were maintained at 28°C with a photoperiod of LD 16:8. Mortality was recorded after 48 h. Poloplus program was used to analyze the dose-response data (POLO-PC, LeOra Software, 1997). Resistance ratios were calculated by dividing LC_50_ values for each strain by that of the Ivf03 strain. If the 95% fiducial limits did not overlap, the spinosad resistance levels of these two strains were considered significantly different (St Leger et al., 1996).

### Construction of NILs in *F. occidentalis*

**Figure 1:**
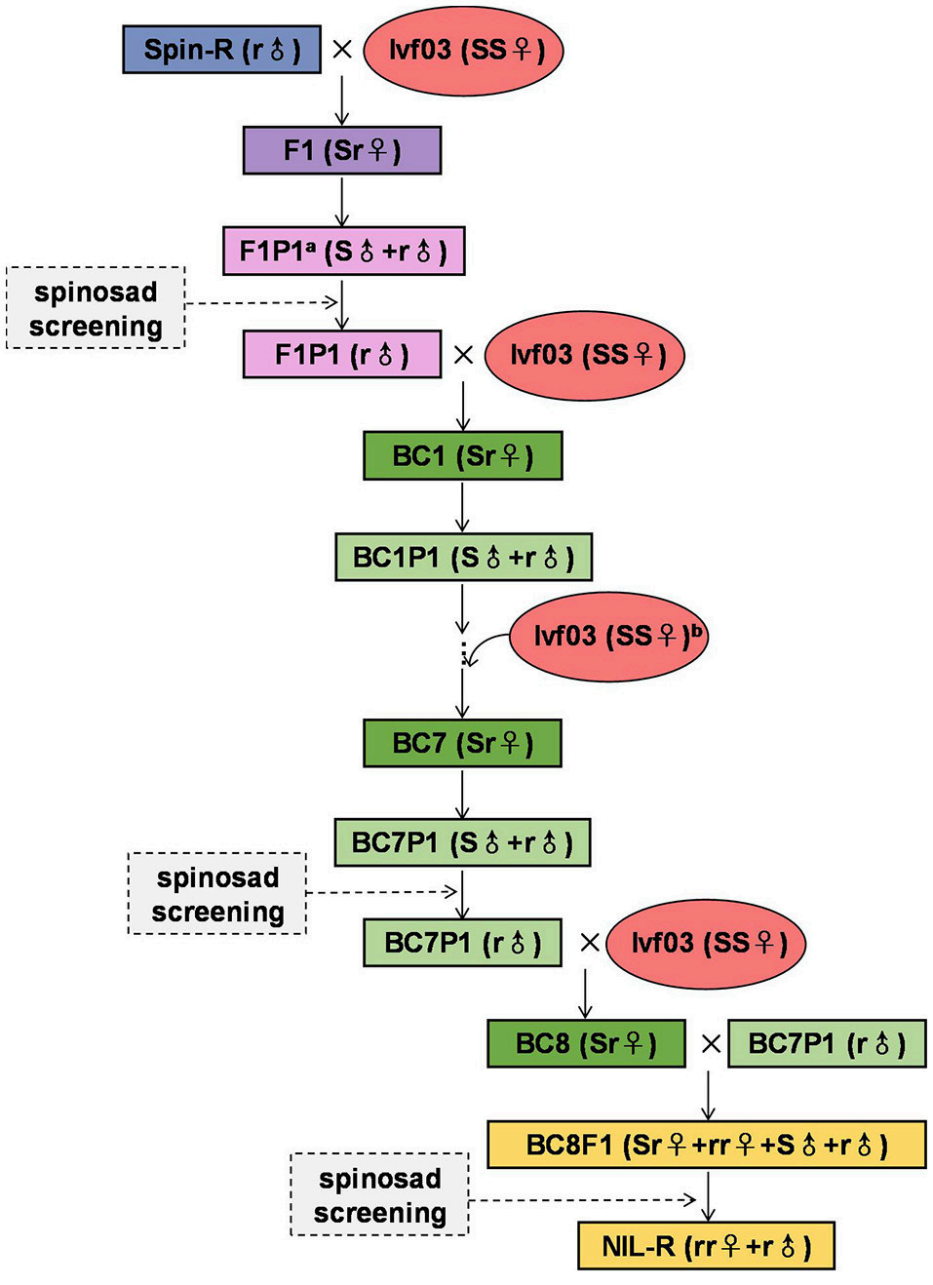
**Crossing strategy with BILs for developing an NIL of *Frankliniella occidentalis* that is resistant to spinosad**. ^*a*^F1P1, offspring of F1 by parthenogenesis; ^*b*^, Add Ivf03 female in every cycle.

Per our ongoing research, *F. occidentalis* female can produce approximately 200 offsprings over the course of its lifetime (QJW, unpublished). For the following experiments, we selected over 500 pupae in each round of crosses. After eclosion, we removed dead pupae and males, and the remaining females (>200) were collected for the parthenogenetic reproduction, which, in theory, would produce over 40,000 individuals. A fine brush pen was used to transfer Ivf03 pupae to 2 mL microcentrifuge tubes (one pupa per tube). After eclosion, females (SS♂) were collected in a glass jar in which they were crossed with Spin-R adult males (r♀). Approximately 100–300 individuals from each sex were used in each backcrosses, and the ratio of male and female was 1:1 (at least 100 pairs of susceptible females and resistant males were mass crossed). Offspring (F1, Sr♀+S♂) were maintained on fresh bean pods until the pupal stage. Pupae from the F1 generation were placed in 2 mL microcentrifuge tubes (one pupa per tube). After eclosion, those F1 virgin females parthenogenetically reproduced their offspring (F1P1, S♂+r♂). The resultant offsprings were treated with 50 mg/L spinosad, a diagnostic concentration that killed all susceptible males but had no impact on resistant males. The survivors (r♂) were then backcrossed with Ivf03 females to obtain the BC1 generation (Sr♀+S♂). This process was repeated for eight cycles to the BC8 generation. Virgin females (Sr♀) from BC8 were then backcrossed with the father generation (BC7P1, r♂) to obtain BC8F1 (Sr♀+rr♀+S♂+r♂). BC8F1 were screened with 50 mg/L spinosad to finally obtain the spinosad-resistant NIL-R strain (rr♀+r♂) (Figure 1).

### DNA extraction

Adults from the two parental strains, the five backcrossed offsprings, and the NIL-R strain were collected and stored individually in a 1.5 mL centrifuge tube at −80°C. Total genomic DNA was extracted using the TIANamp micro DNA kit (TIANGEN Biotech Co., Ltd., Beijing, China) following the manufacturer's protocol. gDNA was extracted from five adult females in each strain, and the concentration ranged between 30 and 50 ng/μL, which was estimated by NanoDrop 2000c spectrophotometer (Thermo Fisher Scientific Inc., Waltham, MA, USA).

### Optimization of ISSR-PCR reaction system

**Figure 2:**
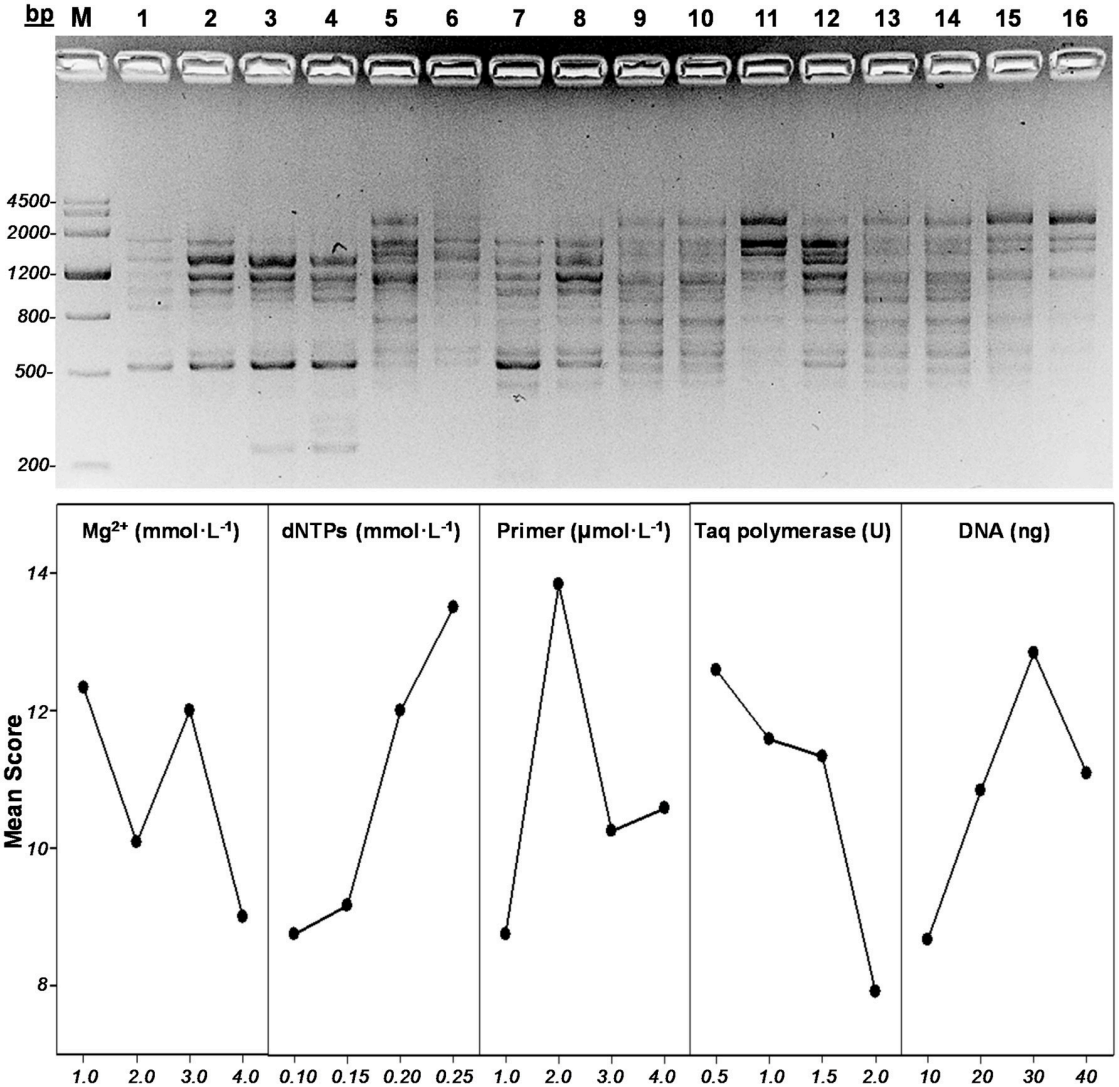
**ISSR analysis. (A)** Electrophoretic profiles of *F. occidentalis* orthogonal ISSR-PCR. Lane 1-16 are the treatments, which involved four levels of the following factors: Mg^2+^ concentration, Taq DNA polymerase content, dNTP concentration, DNA content, and primer concentration. M: Marker III (TIANGEN). **(B)** Scores of five factors plotted against factor level.

Primer sets that amplified the most polymorphic bands, UBC842, was chosen for ISSR-PCR. Furthermore, an orthogonal experiment was designed to optimize ISSR-PCR, including concentrations for Mg^2+^, *Taq* DNA polymerase (CoWin Biosciences, Beijing, China), dNTPs, DNA templates, and primers (Table 1). Each PCR reaction system had a total volume of 20.0 μL that included 2.0 μL of 10x PCR buffer (Mg^2+^ free) (TaKaRa, Dalian, China). Quantity of each ingredient was determined based on the concentration of the stock solution, and distilled water was added to complete the total volume. PCR products were detected by electrophoresis (Figure 2) and were scored using an orthogonal design-direct analysis based on the quantity and brightness of the gel bands. The experiment was conducted and scored three times. IBM SPSS Statistics Version 19 software (SPSS Inc., Chicago, IL) was used for variance analysis. To search for the optimal concentration of individual factors, mean values (Table 2) were plotted against factor levels. Levels with the highest values represented the optimal concentrations of individual factors (Figure 2).

**Table 1:** **L_16_ (4^5^) orthogonal array for optimization of ISSR-PCR**.

**Factor treatment**	**Mg^2+^ (mmol·L^−1^)**	**dNTPs (mmol·L^−1^)**	**Primer (μmol·L^−1^)**	**Taq polymerase (U)**	**DNA (ng)**	**Scores**		
1	1.0	0.10	1.0	0.5	10	7	8	8
2	1.0	0.15	2.0	1.0	20	15	13	15
3	1.0	0.20	3.0	1.5	30	16	14	14
4	1.0	0.25	4.0	2.0	40	12	13	11
5	2.0	0.10	2.0	1.5	40	11	11	14
6	2.0	0.15	1.0	2.0	30	5	6	7
7	2.0	0.20	4.0	0.5	20	12	14	12
8	2.0	0.25	3.0	1.0	10	11	11	13
9	3.0	0.10	3.0	2.0	20	7	6	6
10	3.0	0.15	4.0	1.5	10	8	8	9
11	3.0	0.20	1.0	1.0	40	12	13	11
12	3.0	0.25	2.0	0.5	30	21	23	22
13	4.0	0.10	4.0	1.0	30	9	9	10
14	4.0	0.15	3.0	0.5	40	9	9	8
15	4.0	0.20	2	2.0	10	8	9	7
16	4.0	0.25	1	1.5	20	10	11	9

**Table 2:** **Variance analysis of individual factors affecting ISSR-PCR**.

**Level**	**Mg^2+^ (mmol·L^−1^)**	**dNTPs (mmol·L^−1^)**	**Primer (μmol·L^−1^)**	**Taq polymerase (U)**	**DNA (U)**
Level 1	12.333a	8.750a	8.750a	12.583a	8.667a
Level 2	10.083b	9.167a	13.833b	11.583b	10.833b
Level 3	12.000a	12.000b	10.250c	11.333b	12.833c
Level 4	9.000b	13.500c	10.583c	7.917c	11.083b
*F*-value	34.437	71.262	62.817	56.595	40.024
Rank	5	1	2	3	4

*Different letters (a–c) indicate significant differences*.

### Evaluation of the near-isogenicity by ISSR markers

ISSR analysis was used for the genomic evaluation of the two parental strains, the eight backcross strains, and the final NIL-R strain. Primers (Table 3) that resulted in polymorphic amplifications in *F. occidentalis* were screened from the UBC Primer Set #9 (University of British Columbia Nucleic Acid-Protein Service Unit, UBC Primer Set #9-NAPS Unit). ISSR-PCR was carried out in a thermal cycler (S1000, AB) using a regime of 94°C for 4 min; followed by 35 cycles of 94°C for 40 s, 50–59°C for 40 s, and 72°C for 80 s; and a final extension of 72°C for 10 min. Clear and reproducible amplicon bands were obtained after running the ISSR PCR products in 2% gels, and smeared or weak bands obtained with certain primers were excluded from the ISSR analysis (Zhu et al., 2015). Reproducible bands were scored as 1 (presence) or 0 (absence) for individuals, and matrices generated by each primer sets. The binary matrix was used under Hardy-Weinberg equilibrium to calculate the genetic similarity and distance using POPGEN1.32 (Yeh et al., 1997). The test was repeated three times.

**Table 3:** **Primers used for ISSR amplification**.

**Primer name**	**Sequence (5′-3′)**	**Primer abbreviation**	**Annealing temperature (°C)**
807	AGAGAGAGAGAGAGAGT	(AG) 8T	50
808	AGAGAGAGAGAGAGAGC	(AG) 8C	50
810	GAGAGAGAGAGAGAGAT	(GA) 8T	50
811	GAGAGAGAGAGAGAGAC	(GA) 8C	50
823	TCTCTCTCTCTCTCTCC	(TC) 8C	52
841	GAGAGAGAGAGAGAGAYC	(GA) 8YC^a^	52
842	GAGAGAGAGAGAGAGAYG	(GA) 8YG^a^	52
866	CTCCTCCTCCTCCTCCTC	(CTC) 6	59

a *Y = C/T*.

## Results and discussion

### Development of NIL-R in the parthenogenetically reproduced *F. occidentalis*

A NIL of *F. occidentalis*, which was highly resistant to spinosad (NIL-R), was obtained by eight-round of backcrossing and spinosad screening. The resistance ratios of the F1 and BC3 adult females were <8, respectively, indicating that the resultant progeny had a low level resistance to spinosad. The resistance ratio of NIL-R, however, was >10^4^, demonstrating that NIL-R retained high level of resistance comparable to its parental resistant Spin-R strain (Table 4).

**Table 4:** **Susceptibility of *Frankliniella occidentalis* strains to spinosad**.

**Strain**	***n***	**Slope (SE)**	**LC_50_, 95% FL^a^ (mg/L)**	**χ^2^ (df)^b^**	**RR^c^**
Ivf03	297	1.7 (0.2)	0.072 (0.052–0.096)	0.549 (4)	1
Spin-R	223	2.0 (0.4)	2.7 × 10^3^ (2.0 × 10^3^–4.3 × 10^3^)	1.987 (4)	3.8 × 10^4^
F1	256	1.7 (0.3)	0.46 (0.35–0.66)	1.905 (4)	6.4
BC3	204	1.8 (0.3)	0.52 (0.39–0.70)	0.429 (4)	7.2
NIL-R	255	2.0 (0.3)	2.6 × 10^3^ (2.0 × 10^3^–3.4 × 10^3^)	2.176 (5)	3.6 × 10^4^

a *FL, fiducial limit*.

b *df, degrees of freedom*.

c *RR, resistance ratio. RR = LC_50_ of a strain/LC_50_ of Ivf03*.

In comparison to the backcrossed inbred lines (BILs) used in amphigenetic insects, one major difference was that we obtained resistant males (e.g., F1P1) by parthenogenesis and insecticide screening, while in BILs, self-crossing, insecticide screening and one additional step of males selecting are used to generate resistant males (Zhu et al., 2015). The other difference was the step of BC8 females backcrossing with resistant males from BC7P1, but not self-crossing used in amphigenetic insects. With backcrossing, we obtained the NIL-R resistant females through one hybridization (Figure 1), whereas with self-crossing in a haplo-diploid system; we will need two generations to archive the same goal. Our approach not only saves time but also produces more resistant males.

### Optimization of ISSR-PCR reaction system

The electrophoretic profile of *F. occidentalis* orthogonal ISSR-PCR was shown in Figure 2. According to orthogonal design-direct analysis, the best product had a score of 23, with polymorphic bands, high-definition, and clear background, and the worst product had a score of 1 (Table 1). Based on the *F*-value, the ranking of factors from the most to least effective was dNTPs>primer>Taq polymerase>DNA>Mg^2+^, and the optimal concentration for each factor was 1.0 mmol·L^−1^ Mg^2+^, 0.25 mmol·L^−1^ dNTPs, 2 μmol·L^−1^ primer, 0.5 U of Taq polymerase, and 30 ng DNA template (Table 2, Figure 2). The ranking of factors affecting amplification results is consistent with findings in the multicolored Asian lady beetle, *Harmonia axyridis* Pallas (Guan et al., 2008). The optimal concentration of dNTPs is similar to *S. furcifera* and gypsy moth, *Lymantria dispar* Linnaeus (Chen et al., 2014; Xie et al., 2014). The optimal concentrations of primer and Taq polymerase, however, are different from *H. axyridis, S. furcifera*, and *L. dispar*, suggesting that ISSR-PCR reaction system requires optimization study for each species independently.

### Evaluation of isogenicity

**Figure 3:**
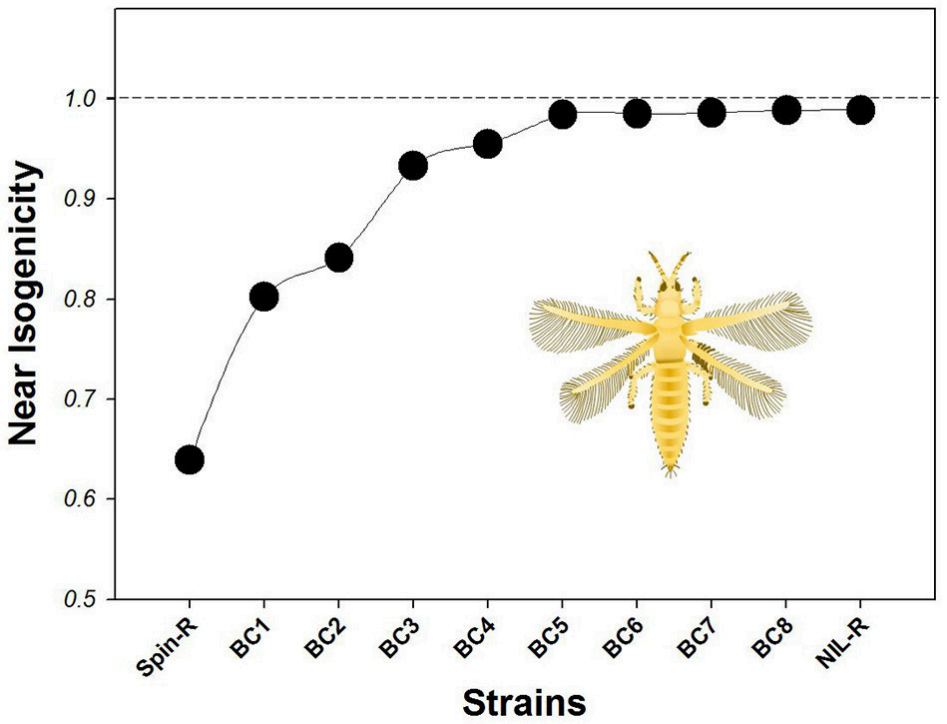
**Genetic similarity of 10 strains based on ISSR polymorphism**. Each point represents the genetic similarity between the corresponding strain and Ivf03. The dash line represents identical genetic background to susceptible parental strain Ivf03.

The genetic similarity between Ivf03 and Spin-R was low (0.6395, Figure 3, Table S1). After one round of crosses and backcrosses, the genetic similarity between Ivf03 and BC1 increased to 0.8024. The isogenicity between Ivf03 and the offspring of backcrosses increased steadily, at the final (8th) round, the genetic similarity between NIL-R and Ivf03 reached 0.9890, indicating a near identical genetic background. Based on the near-isogenicity rating estimated by ISSR marker, NIL-R strain was more similar to its susceptible parent (Ivf03) than to its original resistant parent (Spin-R) while retaining a high level of spinosad resistance (Table 4). These results indicate that the method used here is useful for constructing NILs of *F. occidentalis*.

Suneson (1954) first used the term “Near-isogenic lines” to document the effect of stem rust on the wheat production. Up to date, NILs have been used extensively in economically important crops, including corn, wheat, tomato, and cotton (Wells et al., 1986; Yang et al., 1995; Monforte and Tanksley, 2000; Zhou et al., 2005). Moreover, NILs have been adopted in entomological research to illustrate the genetic underpinnings of insecticide resistance. Fitness cost of insecticide resistance in parthenogenetic insects is direct comparison of fitness between a resistant field population and a susceptible laboratory population (Foster et al., 1997; Bielza et al., 2008). This method, however, overlooks the effect of different genetic background or genes linked to resistance loci, which potentially could have deleterious consequences (Parker, 1991; Bergelson and Purrington, 1996; Haluzik et al., 2004). Clarke and McKenzie (1987) suggested that the genetic background might include specific modifier genes, which could act to reduce fitness cost of the resistant strain. Mu et al. (2005b) have reported that when treated with lambda-cyhalothrin, the respiratory rate of the NIL-R strain was higher than its original resistant strain of the beet armyworm, although they had the same level of resistance. These results demonstrated the biological impacts caused by differences in their genetic backgrounds. Theoretically, phenotypic differences between a pair of NILs can reflect the impacts of the target genes. Therefore, the use of NILs, which differ only in their susceptibility to insecticides would be the most accurate method to characterize the resistance trait, as it allows a precise estimation of the pleiotropic consequences of the resistant mutation by eliminating/mitigating the effect of genetic variance (Jiang et al., 2007; Zhu et al., 2016). Combining with the next generation sequencing (NGS) and QTL mapping, NILs can be a valuable resource to search and map genes associated with resistance at the genome level. By mining the midgut transcriptomes generated from a pair of NILs, a novel ABC transporter was found to be associated with *Bt* resistance in *Plutella xylostella* (Xie et al., 2012; Guo et al., 2015; Zhu et al., 2015, 2016). Field evolved spinosad resistance has been documented in several insect pest species (Baxter et al., 2010; Hsu et al., 2012; Puinean et al., 2013; Hou et al., 2014). The mechanistic study of spinosad resistance in *F. occidentalis* will be greatly facilitated by these newly established resistant and susceptible NILs.

Construction methods of NILs in plants and amphigenetic insects are basically the same because of the commonality in their bisexual production system. In parthenogenetically reproduced animals, however, these conventional strategies have failed, leaving a significant gap in the mechanistic study of resistance in these animals. Parthenogenesis occurs predominantly among insects, including Apoidea, Chalcidoidea, Coccoidea, Thripidae, Aleyroidae, and two Scolytidae-the bark and ambrosia beetles (Darlington, 1937; Whiting, 1945; Takenouchi and Takagi, 1967; White, 1977). Parthenogenesis plays an important role in the range distribution and population augmentation in some insect species. Compared with amphigenetic insects, parthenogenetic insects can occupy certain niche more effectively (Kearney, 2005). Because parthenogenesis allows insects to exploit new resources while avoiding the constraints of finding a mate, so they are able to survive in a wide range of environmental conditions, which is essential for successful colonization and invasiveness (Davis, 2009; Liebhold et al., 2016). However, Domes et al. (2007) reported that parthenogenetic oribatid soil mites had a greater fitness cost (e.g., reduced egg production) associated with the resource depletion than its sexual counterparts, suggesting that parthenogenetic species tend to have advantages over sexual ones in constant than variable environments. This, at least partially, explains why parthenogenesis is much common among agricultural pests than other non-pest species, as agricultural environments are stable and resource-abundant.

Phloem-feeding arthropods, including aphids, whiteflies, thrips, and spider mites, reproduce both asexually and sexually. By rotating these two reproductive strategies, they can breed rapidly under not only stable conditions, but also changing environments (Kanbe and Akimoto, 2009). This unique reproductive strategy, coupled with the ineffectiveness of *Bt* toxins (Raps et al., 2001; Dutton et al., 2002) and the development of insecticide resistance (Walling, 2008) make phloem-feeding arthropods one of the most challenging group of pests to control. Here we established the first NILs in a parthenogenetically reproduced thrips species. By developing this toolset, we are able to segregate individual resistance and facilitate the mechanistic study of insecticide resistances in phloem-feeding arthropods.

## Author contributions

QW, GY, YW, and BH. conceived and designed the experiment; GY. performed the experiment; GY, YW, XL, and XZ. analyzed the data; BX, WX, SW, and YZ. contributed reagents/materials/analysis tools; GY. drafted the paper. QW and XZ. edited the manuscript and supervised the entire project.

## Funding

This work was supported by grants from the National Science and Technology support task (2012BAD19B06), the Natural Science Foundation of China (31371965, 31572037), the Beijing Key Laboratory for Pest Control and Sustainable Cultivation of Vegetables, and the Science and Technology Innovation Program of the Chinese Academy of Agricultural Sciences (AAS-ASTIP-IVFCAAS).

### Conflict of interest statement

The authors declare that the research was conducted in the absence of any commercial or financial relationships that could be construed as a potential conflict of interest.
