# Supplementary material for: Development of Near-Isogenic Lines in a Parthenogenetically Reproduced Thrips Species, Frankliniella occidentalis
Source: Front Physiol. 2017 Mar 13;8:130. doi: 10.3389/fphys.2017.00130 (PMC5346592; doi:10.3389/fphys.2017.00130)
Supplement: Supplementary file 1 [file Table1.docx]

**Supplementary Materials**

**Table S1. Genetic similarity among eleven *F. occidentalis* strains based on ISSR polymorphism**

|  | Ivf03 | Spin-R | BC1 | BC2 | BC3 | BC4 | BC5 | BC6 | BC7 | BC8 | NIL-R |
| --- | --- | --- | --- | --- | --- | --- | --- | --- | --- | --- | --- |
| Ivf03 | 1.0000 |  |  |  |  |  |  |  |  |  |  |
| Spin_R | 0.6395 | 1.0000 |  |  |  |  |  |  |  |  |  |
| BC1 | 0.8024 | 0.9106 | 1.0000 |  |  |  |  |  |  |  |  |
| BC2 | 0.8413 | 0.8755 | 0.9625 | 1.0000 |  |  |  |  |  |  |  |
| BC3 | 0.9331 | 0.7860 | 0.9181 | 0.9420 | 1.0000 |  |  |  |  |  |  |
| BC4 | 0.9553 | 0.7262 | 0.8713 | 0.9094 | 0.9569 | 1.0000 |  |  |  |  |  |
| BC5 | 0.9843 | 0.6573 | 0.8183 | 0.8587 | 0.9418 | 0.9614 | 1.0000 |  |  |  |  |
| BC6 | 0.9851 | 0.6450 | 0.8043 | 0.8533 | 0.9349 | 0.9603 | 0.9855 | 1.0000 |  |  |  |
| BC7 | 0.9865 | 0.6226 | 0.7847 | 0.8309 | 0.9230 | 0.9491 | 0.9851 | 0.9937 | 1.0000 |  |  |
| BC8 | 0.9886 | 0.6243 | 0.7912 | 0.8298 | 0.9292 | 0.9461 | 0.9852 | 0.9873 | 0.9944 | 1.0000 |  |
| NIL-R | 0.9890 | 0.6217 | 0.7893 | 0.8250 | 0.9261 | 0.9507 | 0.9893 | 0.9823 | 0.9891 | 0.9932 | 1.0000 |
